# Comparing the Diagnostic Characteristics of Neuropathy Questionnaires in Adults With Obesity

**DOI:** 10.1111/jns.70163

**Published:** 2026-08-21

**Authors:** Nicholas Heaton, Evan L. Reynolds, Fallon Koenig, Lavanya Muthukumar, Merel Huijg, Noor E. Taams, Pieter A. van Doorn, Brian C. Callaghan

**Affiliations:** ^1^ Department of Neurology University of Michigan Ann Arbor Michigan USA; ^2^ Department of Epidemiology and Biostatistics Michigan State University East Lansing Michigan USA; ^3^ Department of Neurology Erasmus University Medical Center Rotterdam the Netherlands; ^4^ Department of Epidemiology Erasmus University Medical Center Rotterdam the Netherlands

**Keywords:** diagnostic accuracy, peripheral neuropathy, questionnaire

## Abstract

**Aim:**

To compare the diagnostic characteristics of three peripheral neuropathy (PN) patient‐completed questionnaires.

**Methods:**

We recruited participants with obesity from a previously completed randomized controlled trial. Patient‐completed PN questionnaires included the Michigan Neuropathy Screening Instrument questionnaire (MNSIq), the Diabetic Neuropathy Symptom (DNS) score, and the Erasmus Polyneuropathy Symptom Score (E‐PSS). PN was defined using the Toronto consensus definition for confirmed neuropathy. Secondary PN definitions included probable neuropathy and definitions based on skin biopsy, nerve conduction studies, and clinical scales. Diagnostic accuracy was determined using the area under the receiver operating characteristic curve (AUC), and significant differences in AUC were assessed with DeLong's test. Potential diagnostic thresholds and corresponding predictive values and likelihood ratios were calculated.

**Results:**

We enrolled 59 participants (mean [SD] age: 53 [7] years, 76% female, 81% White), and 20.3% had neuropathy. E‐PSS had the largest AUC (95% confidence interval) for confirmed neuropathy (0.84, 0.71–0.97), followed by MNSIq (0.76, 0.63–0.90) and the DNS (0.72, 0.54–0.90), but these differences were not significant (*p* > 0.05). No significant differences were seen for secondary PN definitions (all *p* > 0.05). All three questionnaires were highly correlated with one another (*r* range: 0.70–0.72, all *p* < 0.01), and diagnostic thresholds were estimated.

**Interpretation:**

All three patient‐completed questionnaires displayed good diagnostic accuracy for PN and were highly correlated. Our findings suggest that questionnaires can be used to determine PN in large epidemiological studies and might be useful as screening instruments in clinical care.

## 1 Introduction

Peripheral neuropathy (PN) is estimated to affect 13.5% of the US population aged 40 and over [1] and is associated with depression, reduced quality of life and mobility, and all‐cause mortality [2]. Obesity is the second leading metabolic risk factor for PN, after diabetes [3, 4]. In addition, as the prevalence of obesity continues to increase, with 1.5 billion adults worldwide projected to have obesity by 2035 [5], it is likely that obesity‐related PN will also increase [6]. A PN diagnosis requires comprehensive history and neurological examination. However, neurologists' diagnoses are not always feasible for epidemiological studies given the costs and availability of neurologists. Therefore, simple patient‐reported questionnaires are ideal to assess for PN in large population‐based studies. Similarly, questionnaires may be ideal clinical screening instruments, as they can be rapidly collected, have minimal associated costs, and are noninvasive. Two commonly used questionnaires used to assess PN are the Michigan Neuropathy Screening Instrument questionnaire (MNSIq) [7] and the Diabetic Neuropathy Symptom (DNS) score [8]. In previous validation studies, MNSIq and DNS had sensitivities of 0.40 and 0.79 and specificities of 0.92 and 0.78, respectively [8, 9]. More recently, the Erasmus Polyneuropathy Symptom Score (E‐PSS) was developed as an additional screening tool for PN [10].

Despite their utility in epidemiological research, few studies have compared the diagnostic accuracy of these questionnaires to gold standard definitions, including neurological history and examinations, and/or confirmatory testing. Furthermore, only MNSIq has been validated solely among adults with obesity: our two studies of adults with obesity (*n* = 120 and *n* = 140) found that MNSIq had good diagnostic accuracy for PN (area under the receiver operating characteristic [ROC] curve [AUC] range: 0.75–0.76), defined using the Toronto consensus definition for probable polyneuropathy [11, 12]. Similarly, MNSIq had an AUC of 0.73 for determining PN in a cohort of 1184 individuals with Type 1 diabetes [9]. Only one study characterized the diagnostic accuracy of the DNS, which found an AUC of 0.74 for determining PN defined using nerve conduction studies (NCSs) among 214 persons with diabetes [13]. Similarly, one study demonstrated that the E‐PSS had an AUC of 0.95 for distinguishing PN in 140 patients with chronic idiopathic axonal polyneuropathy compared to 96 controls [10]. However, no studies have investigated the head‐to‐head diagnostic accuracy of these three different questionnaires, and none have compared and validated the questionnaires in adults with obesity. Thus, which questionnaire is optimal to define PN is unclear.

To address this knowledge gap, we compared the discriminatory capabilities of the E‐PSS, DNS, and MNSIq questionnaires to a gold standard PN assessment incorporating a neuromuscular physician's standardized assessment and confirmatory testing with NCS and skin biopsies in a population with obesity. We also calculated predictive values for PN based on multiple diagnostic thresholds to propose possible screening strategies for PN. We also assessed the correlations among DNS, E‐PSS, and MNSIq.

## 2 Materials and Methods

### 2.1 Population and Study Design

This study is a secondary analysis of participants enrolled in a randomized clinical trial (clinicaltrials.gov: NCT03617185) [12]. Recruitment took place from October 2018 to July 2022 from three surgical weight loss clinics in southeastern Michigan, USA. Inclusion criteria for the initial study required participants to be at least 40 years of age and attend a bariatric surgery clinic. In addition, a body mass index (BMI) of at least 35 kg/m^2^ with a weight‐related comorbidity or at least 40 kg/m^2^ without comorbidities. Exclusion criteria included current smoking, a failed exercise stress test, use of walking assist devices, and other criteria previously described [12]. During the initial randomized controlled trial, participants were followed for 24 months. PN outcomes for this present study were completed at the 24‐month timepoint for the initial randomized controlled trial, and recruitment for the present study began after the completion of the randomized controlled trial.

Following the completion of the 24‐month randomized controlled trial, we completed an ancillary study to compare the diagnostic accuracy of MNSIq, DNS, and E‐PSS. Specifically, we contacted study individuals from February 2025 to June 2025 by email and then by telephone to recruit for the present study. This separate recruitment occurred after the completion of the original randomized controlled trial. We obtained informed consent for the present study separate from the initial randomized controlled trial. Inclusion criteria for the present study required completion of the initial 24‐month randomized controlled trial and completion of the three questionnaires. Participants who consented to filling out the three questionnaires for the present study completed them on the same day, independently and remotely, on their personal devices or on paper, and submitted responses by mail. The participants completed the MNSIq first, then the DNS, and last the E‐PSS. The present study was approved by the University of Michigan Medical School Institutional Review Board. No intervention occurred between the 24‐month outcome visit and the administration of the questionnaires.

### 2.2 PN Screening Questionnaires

MNSIq is a self‐administered 15‐item questionnaire that can be answered with either *yes* or *no* [9]. Each *yes* is worth one point, with the exception that answering *no* to Questions 7 and 13 adds a point. The score range for MNSIq is 0–15.

DNS is a shorter four‐item questionnaire that can be answered with either *yes* or *no* [8]. The score range for DNS is 0–4.

E‐PSS contains six questions, and each question can be answered on the ordinal scale, *never*, *sometimes*, or *(almost) continuously* [10]. The weighted sum score range for E‐PSS is 0–14.

### 2.3 Gold Standard PN Definition

We used the Toronto consensus definition for confirmed neuropathy as our gold standard PN diagnosis [14]. This definition required the presence of at least one of the following: symptoms consistent with distal symmetrical polyneuropathy, an abnormal sensory exam, or decreased or absent deep tendon reflexes as determined by a neuromuscular specialist (Toronto consensus definition of probable neuropathy). Participants were also required to have either abnormal NCS results or abnormal intraepidermal nerve fiber density (IENFD) at the distal leg. NCS abnormality was determined by at least one abnormality in two different nerves (tibial, peroneal, or sural nerves). Peroneal and tibial abnormality was evaluated with the following metrics: amplitude, *F* wave, and distal motor latency (DML); in addition, peroneal abnormality was also assessed with conduction velocity. Sural abnormality was measured by amplitude and peak latency [15]. Abnormalities in NCS measurements were confirmed using the 3rd/97th percentiles of previously published normative values [16]. IENFD was collected as previously described [12]. An IENFD abnormality at the distal leg was confirmed using the fifth percentile of previously published age‐ and sex‐specific normative values [17].

### 2.4 Secondary PN Diagnosis

Secondary PN outcomes included definitions based on NCS (as above), IENFD (as above), abnormal NCS alone, abnormal IENFD alone, and the Toronto consensus definition for probable neuropathy (two or more of the following: symptoms consistent with distal symmetrical polyneuropathy, abnormal sensory exam, decreased or absent deep tendon reflexes), and based on physical examination scales, including Utah Early Neuropathy Screening (UENS), modified Toronto Clinical Neuropathy Scale (mTCNS), and Michigan Neuropathy Screening Instrument exam (MNSIe). PN was defined as a UENS score greater than or equal to four [18, 19], an mTCNS score greater than or equal to three [20], and an MNSIe score greater than or equal to 2.5 [7].

### 2.5 Statistical Analysis

Continuous variables were reported as mean (SD) and categorical variables as counts (%) to summarize participant demographic characteristics according to confirmed neuropathy status. A series of univariate logistic regression models were fit for each PN outcome as a function of MNSIq, DNS score, and the E‐PSS score separately. The discriminatory accuracy of each questionnaire was determined by assessing the AUC of the respective ROC curves. DeLong's method was used to determine 95% confidence intervals (CIs). DeLong's test for difference in AUCs was used to compare the three separate models for each outcome. We proposed potential diagnostic thresholds for confirmed neuropathy using the optimal Youden's *J* index, previously established thresholds for each instrument [8, 9], and constrained 90% sensitivity and specificity. There was no established threshold readily available for the E‐PSS questionnaire, and we thus did not include established threshold metrics for this study [10]. In each scenario, we calculated sensitivity, specificity, positive likelihood ratio (LR+), positive predictive value (PPV), and negative predictive value (NPV) based on the respective thresholds. Pairwise Spearman's correlation coefficients were calculated to assess the relationships between the three questionnaire scores.

A complete case analysis was carried out to handle missing responses or outcomes and ensure the models were fit using the same data. Statistical significance was determined using a *p* value threshold of 0.05. All analyses were completed using R software version 4.5.0.

## 3 Results

### 3.1 Study Participants

**FIGURE 1:**
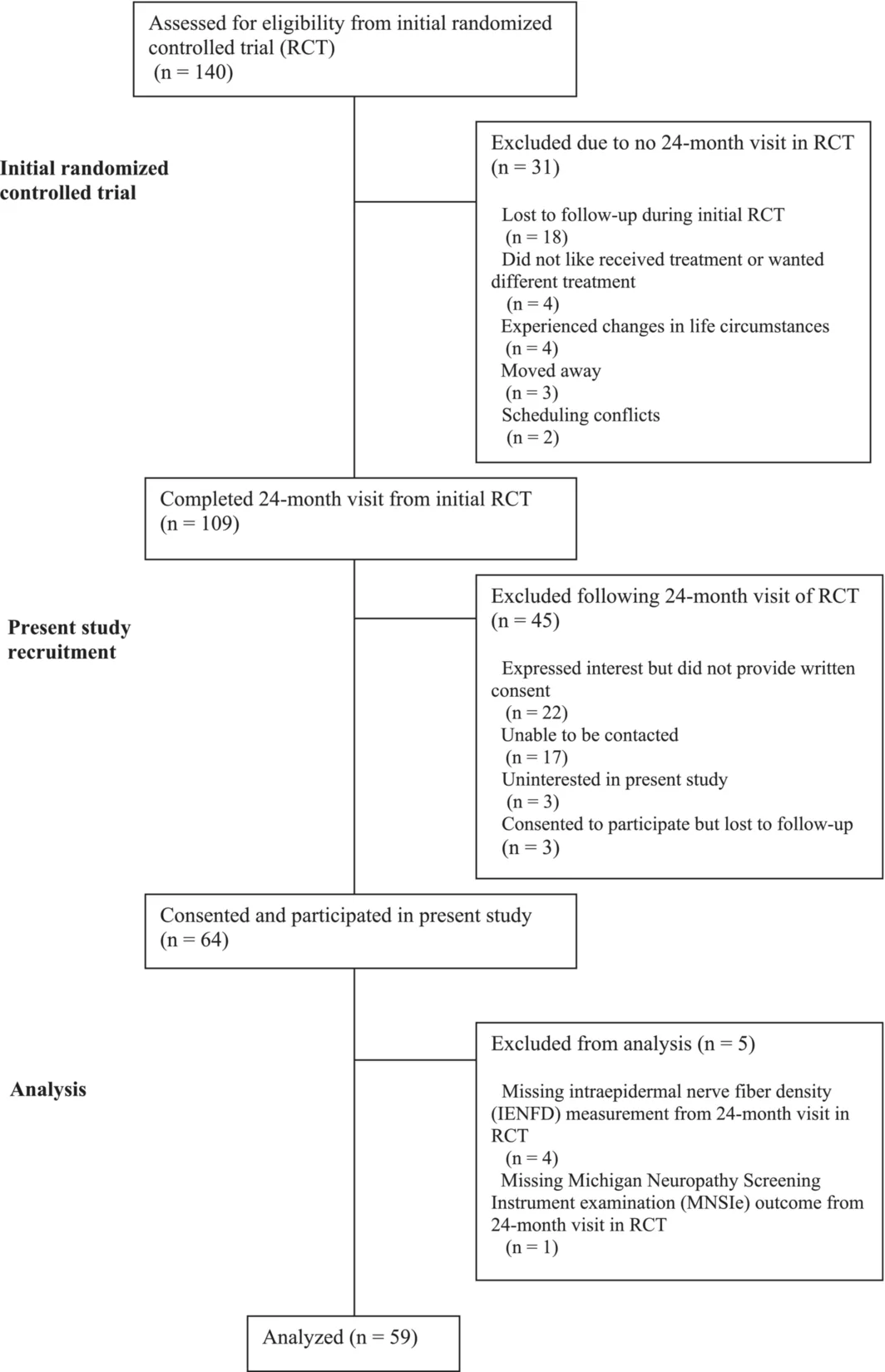
Study eligibility and recruitment diagram.

Of the 140 participants enrolled in the initial randomized controlled trial, 109 completed 24 months of follow‐up: 18 were lost to follow‐up, four had changes in life circumstances, four did not like the treatment received or wanted a different treatment, three moved away, and two had scheduling conflicts (Figure 1). Of those 109 participants who had completed the 24‐month visit from the randomized controlled trial, 64 consented to enroll in this ancillary study and had completed all items within the 3 questionnaires. Of the 109 that completed the 24‐month visit, 22 were interested but never provided written consent for the present study, 17 were unable to be contacted, three were not interested, and 3 had consented to participate but were then lost to follow‐up. Four (6.3%) were missing IENFD measurements from the 24‐month timepoint, and one (1.6%) was missing MNSIe results from the 24‐month timepoint, resulting in 59 participants with complete data. The median (IQR) follow‐up time between the neuromuscular examination visit at the 24‐month timepoint and ancillary survey completion was 2.1 (1.7, 3.8) years.

The mean (SD) age at the time of the neuromuscular examination was 53 (7) years; 45 (76%) were female, and most participants were White (81%) (Table 1). In addition, the mean (SD) BMI was 38 (8) kg/m^2^. Furthermore, 8 (14%) participants had diabetes and 15 (25%) had prediabetes, as defined by the 2025 American Diabetes Association Standards of Care [21].

**TABLE 1:** Demographic characteristics of study participants stratified by presence of neuropathy.

Characteristic	Overall (*N* = 59)	No neuropathy (*N* = 47)	Neuropathy (*N* = 12)
*n* (%)			
Mean age (SD) (years)	53 (7)	52 (7)	59 (8)
Sex			
Female	45 (76%)	39 (83%)	6 (50%)
Race			
Bi/multiracial	2 (3.4%)	2 (4.3%)	0 (0%)
Black	8 (14%)	6 (13%)	2 (17%)
White	48 (81%)	39 (83%)	9 (75%)
Middle Eastern	1 (1.7%)	0 (0%)	1 (8.3%)
Smoking status			
Never smoker	39 (66%)	30 (64%)	9 (75%)
Education status			
High school graduate or GED	4 (6.8%)	3 (6.4%)	1 (8.3%)
Vocational college or some college	17 (29%)	12 (26%)	5 (42%)
College degree	26 (44%)	24 (51%)	2 (17%)
Professional or graduate degree	12 (20%)	8 (17%)	4 (33%)
Diabetes status			
Prediabetes	15 (25%)	12 (26%)	3 (25%)
Diabetes	8 (14%)	4 (8.5%)	4 (33%)
Mean BMI (SD) (kg/m^2^)	38 (8)	38 (9)	38 (6)

We found 12 (20.3%) of the 59 participants had confirmed neuropathy at the 24‐month timepoint neuromuscular examination visit. In addition, 8 (13.6%) had confirmed neuropathy using NCS alone, 9 (15.3%) had confirmed neuropathy using IENFD alone, 10 (16.9%) of the 59 participants had abnormal NCS measurements, 12 (20.3%) had abnormal IENFD, 17 (28.8%) had probable neuropathy, 23 (39.0%) had abnormal UENS, 22 (37.3%) had abnormal mTCNS, and 11 (18.6%) had abnormal MNSIe.

The median (IQR) scores for the E‐PSS, MNSIq, and DNS were 1.0 (0.0, 2.0), MNSIq of 2.0 (0.0, 3.0), and DNS of 0.0 (0, 1.0). The median (IQR) scores of E‐PSS, MNSIq, and DNS for participants with confirmed neuropathy were 2.0 (1.5, 4.0), 3.0 (2.5, 5.5), and 2.0 (0.0, 3.0), respectively. Participants without confirmed neuropathy had average (IQR) scores of 0.0 (0.0, 1.0) for E‐PSS, 2.0 (0.0, 3.0) for MNSIq, and 0.0 (0.0, 1.0) for DNS.

### 3.2 Diagnostic Accuracy for Confirmed Neuropathy

**FIGURE 2:**
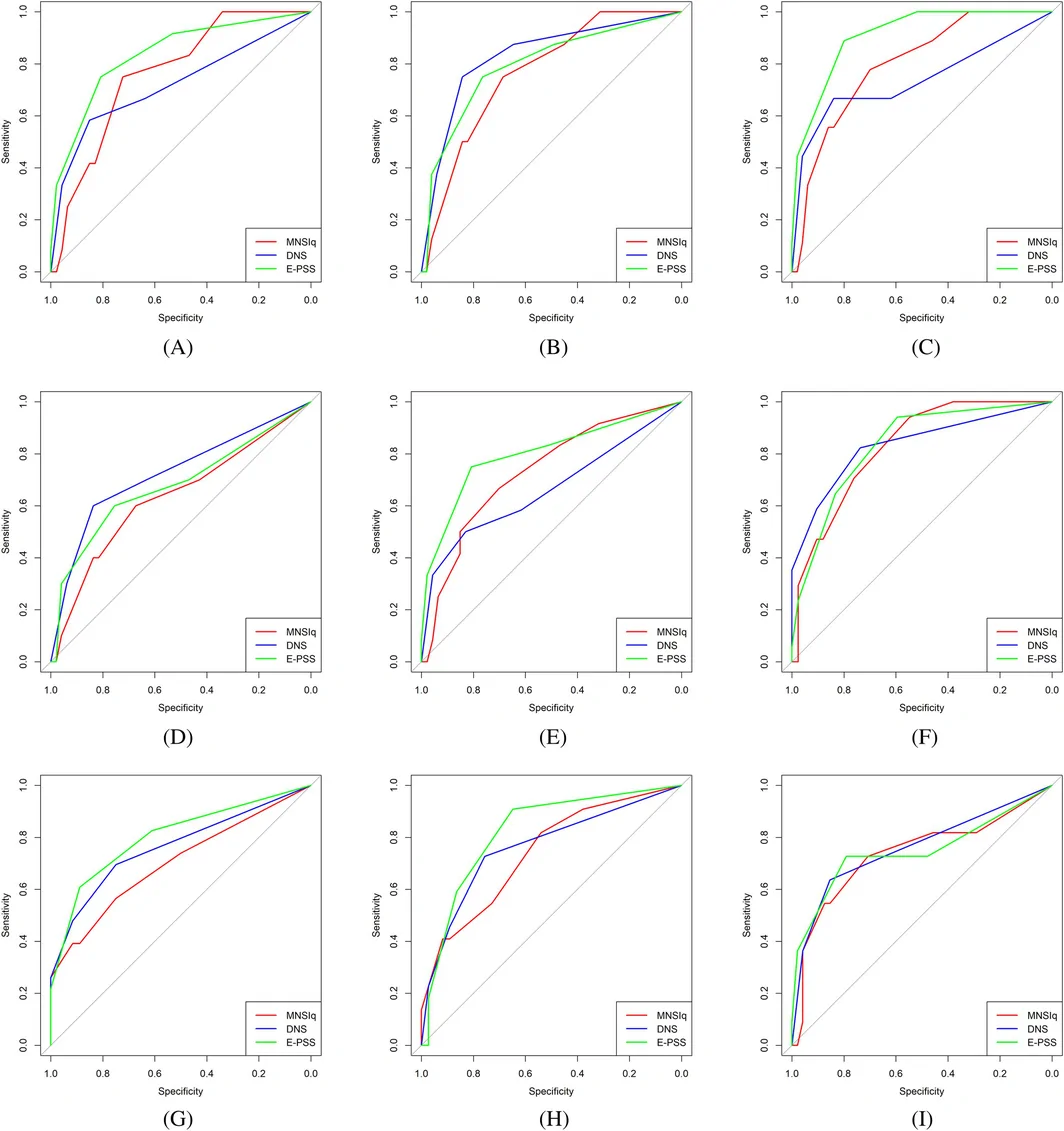
Receiver operator characteristic (ROC) curves for MNSIq, DNS, and E‐PSS questionnaires for (A) gold standard confirmed neuropathy; (B) NCS‐based confirmed neuropathy; (C) IENFD‐based confirmed neuropathy; (D) NCS abnormality; (E) IENFD abnormality; (F) probable neuropathy; (G) UENS; (H) mTCNS; and (I) MNSIe. (A) Gold standard confirmed neuropathy (12 [20.3%] participants had confirmed neuropathy). (B) NCS‐based confirmed neuropathy (eight [13.6%] participants had NCS‐based confirmed neuropathy). (C) IENFD‐based confirmed neuropathy (9 [15.3%] participants had IENFD‐based confirmed neuropathy). (D) NCS (10 [16.9%] participants had abnormal NCS). (E) IENFD (12 [20.3%] participants had abnormal IENFD). (F) Probable neuropathy (17 [28.8%] participants had probable neuropathy). (G) UENS (23 [39.0%] had abnormal UENS). (H) mTCNS (22 [37.3%] had abnormal mTCNS). (I) MNSIe (11 [18.6%] participants had abnormal MNSIe).

We found that E‐PSS had the highest AUC (95% CI) for confirmed PN (0.84, 0.71–0.97), followed by MNSIq (0.76, 0.63–0.90) and then DNS (0.72, 0.54–0.90) (Table 2; Figure 2). However, there were no significant differences between the three questionnaires (E‐PSS vs. MNSIq: *p* = 0.33; E‐PSS vs. DNS: *p* = 0.11; MNSIq vs. DNS: *p* = 0.62).

**TABLE 2:** Diagnostic accuracy of neuropathy questionnaires.

Outcome	MNSIq AUC (95% CI)	DNS AUC (95% CI)	E‐PSS AUC (95% CI)
Confirmed neuropathy	0.76 (0.63, 0.90)	0.72 (0.54, 0.90)	0.84 (0.71, 0.97)
NCS‐based confirmed neuropathy	0.76 (0.61, 0.93)	0.83 (0.67, 0.99)	0.80 (0.62, 0.98)
IENFD‐based confirmed neuropathy	0.80 (0.65, 0.94)	0.74 (0.52, 0.95)	0.91 (0.83, 0.99)
Abnormal NCS	0.64 (0.43, 0.84)	0.73 (0.54, 0.91)	0.68 (0.47, 0.89)
Abnormal IENFD	0.73 (0.57, 0.89)	0.66 (0.48, 0.85)	0.80 (0.64, 0.96)
Probable neuropathy	0.83 (0.72, 0.93)	0.84 (0.72, 0.95)	0.83 (0.73, 0.94)
UENS	0.70 (0.56, 0.84)	0.76 (0.64, 0.89)	0.80 (0.69, 0.92)
mTCNS	0.74 (0.61, 0.87)	0.76 (0.64, 0.89)	0.82 (0.72, 0.93)
MNSIe	0.74 (0.54, 0.93)	0.76 (0.58, 0.94)	0.75 (0.55, 0.95)

Abbreviations: IENFD, abnormal intraepidermal nerve fiber density; MNSIe, abnormal Michigan Neuropathy Screening Instrument exam; mTCNS, modified Toronto Clinical Neuropathy Score; NCS, abnormal nerve conduction studies; UENS, abnormal Utah Early Neuropathy Scale.

The thresholds required for optimal Youden's J index were MNSIq ≥ 3, DNS ≥ 2, and E‐PSS ≥ 2 (Table 3). At these thresholds, the E‐PSS had a sensitivity of 0.75, specificity of 0.81, LR+ of 3.93, PPV of 0.50, and NPV of 0.93. MNSIq had a sensitivity of 0.75, specificity of 0.72, LR+ of 2.68, PPV of 0.41, and NPV of 0.92. Finally, DNS had a sensitivity of 0.58, a specificity of 0.85, an LR+ of 3.91, a PPV of 0.50, and an NPV of 0.89. We also calculated these metrics for previously established thresholds and constrained 90% sensitivities and specificities. However, when constrained to 90% sensitivity and specificity, we were unable to keep the opposite metric (specificity or sensitivity) at a high value (Table 3).

**TABLE 3:** Predictive values for potential diagnostic thresholds using questionnaires.

Instrument	Threshold method	Sensitivity	Specificity	LR+	PPV	NPV	Optimal threshold
MNSIq	Optimal Youden's *J*	0.75	0.72	2.68	0.41	0.92	≥ 3
	Established threshold	0.42	0.83	2.45	0.39	0.85	≥ 4
	Constrained 90% sensitivity	0.90	0.34	1.52	0.28	1.00	≥ 1
	Constrained 90% specificity	0.25	0.90	3.91	0.50	0.83	≥ 6
DNS	Optimal Youden's *J*	0.58	0.85	3.91	0.50	0.89	≥ 2
	Established threshold	0.67	0.64	1.84	0.32	0.88	≥ 1
	Constrained 90% sensitivity	0.90	0.00	1.00	0.20	NA	≥ 0
	Constrained 90% specificity	0.33	0.90	6.66	0.67	0.85	≥ 3
E‐PSS	Optimal Youden's *J*	0.75	0.81	3.93	0.50	0.93	≥ 2
	Constrained 90% sensitivity	0.90	0.53	1.96	0.33	0.96	≥ 1
	Constrained 90% specificity	0.33	0.90	15.86	0.80	0.85	≥ 4

*Note:* The NPV for DNS at a 90% constrained sensitivity is not estimable as no true negatives were identified at the specified threshold. The established threshold for E‐PSS was not readily available and was thus not included in this study [10].

Abbreviations: LR+, positive likelihood ratio; NPV, negative predictive value; PPV, positive predictive value.

### 3.3 Diagnostic Accuracy for Secondary Outcomes

The questionnaire with the highest diagnostic accuracy (AUC, 95% CI) for confirmed neuropathy using NCS was DNS (0.83, 0.67–0.99); IENFD‐based confirmed neuropathy was E‐PSS (0.91, 0.83–0.99); abnormal NCS was DNS (0.73, 0.54–0.91); for abnormal IENFD, it was E‐PSS (0.80, 0.64–0.96); and for probable neuropathy, it was DNS (0.84, 0.72–0.95). The questionnaire with the highest diagnostic accuracy for abnormal MNSIe was DNS (0.76, 0.58–0.94); for abnormal mTCNS, it was E‐PSS (0.82, 0.72–0.93); and for abnormal UENS, it was E‐PSS (0.80, 0.69–0.92). However, we found no significant differences in AUCs between the three questionnaires for any secondary outcomes (all *p* > 0.05) (Table 2; Figure 2).

### 3.4 Correlation Between Questionnaire Responses

The E‐PSS, DNS, and MNSIq questionnaires were all significantly correlated with one another (*r* = 0.70–0.72, *p* < 0.01 for all three correlations).

## 4 Discussion

We performed the first head‐to‐head comparison of PN questionnaires to a gold standard PN definition, including neurologic history, examination, and confirmatory testing. We found E‐PSS had the highest diagnostic accuracy (AUC = 0.84), followed by MNSIq (AUC = 0.76) and DNS (AUC = 0.72), although no statistically significant differences were observed. Furthermore, the diagnostic accuracy of the three questionnaires was similar, regardless of how PN was defined using neuromuscular assessment and/or confirmatory testing or with physician examination scales. Correlations between the three questionnaires were also very high (> 0.7). Thus, our findings indicate that simple PN screening questionnaires have high diagnostic accuracy for identifying PN in populations with obesity and can be reliably used to identify PN in epidemiologic studies. These questionnaires may also have potential as clinical screening instruments, and potential thresholds with corresponding sensitivity, specificity, LR+, PPV, and NPV were calculated for each questionnaire. However, future studies are needed to address their precise role in clinical care and in populations without obesity.

Although the resulting AUCs were not statistically different, the E‐PSS had the highest diagnostic accuracy, followed by MNSIq, and then DNS. Furthermore, the E‐PSS had the largest AUC for five out of the nine outcomes. However, larger studies are needed to determine if E‐PSS is significantly better than the MNSIq and DNS given the limited power of the current study. The resulting AUCs in the present study were remarkably similar to past studies evaluating the MNSIq and DNS. Specifically, for MNSIq, the observed AUC, 0.76, was similar to the three previous studies that determined diagnostic accuracy (AUCs ranging from 0.73–0.76) [9, 11, 12]. Furthermore, the AUC observed for DNS, 0.72, was similar to one previous study (AUC = 0.74) [13]. While our AUC estimate for the E‐PSS was lower than previously reported (0.84 compared to 0.95) [10], the E‐PSS remains the questionnaire with the highest reported diagnostic accuracy. The three questionnaires were also highly correlated (*r* range: 0.70–0.72), suggesting they yield comparable assessments among respondents. These consistent findings indicate that all three questionnaires could be used to determine neuropathy status with high diagnostic accuracy for epidemiologic studies. However, given that no questionnaire AUC is above 0.84, except the E‐PSS for IENFD‐based confirmed neuropathy, questionnaires might have a ceiling on their diagnostic accuracy. Alternatively, these questionnaires may have artificially lower accuracy because PN may have continued to progress during the 2.1 years between the end of the original randomized controlled trial and the assessment of the questionnaires for the present study. Nevertheless, these questionnaires are unlikely to replace neurological history and examinations needed to make a diagnosis in the clinic settings.

Despite the comparable diagnostic accuracy and high correlations, there are differences in the design of the three questionnaires. Specifically, MNSIq is the longest of the 3, with 15 items, and scoring MNSIq is potentially confusing. There are negatively worded items in MNSIq, as answering “no” to these adds to the patient's score while answering “yes” to the other items adds to the overall score. The utility of negatively worded items has been debated when designing questionnaires, as they may not protect against passive acceptance behaviors among respondents [22]. In addition, two of the 15 items were designed to record vascular but not neuropathy symptoms. The original scoring for MNSIq did not include these two questions, but more recent scoring suggests that these should be included [7, 9]. Meanwhile, DNS and E‐PSS are shorter with four and six items, respectively. Furthermore, these two questionnaires do not have items that are negatively worded or designed to assess non‐neuropathic symptoms, making scoring and implementation more straightforward compared to MNSIq. In addition, these two surveys only ask about symptoms, while MNSIq includes an item directly asking patients if they have been diagnosed with diabetic neuropathy, which can introduce unnecessary bias from the respondents [23]. While MNSIq can be more confusing, it has been validated in several language and cultural translations, providing more widespread accessibility [24, 25, 26, 27]. Thus, even if the E‐PSS may have greater diagnostic accuracy, in some settings, a shorter questionnaire (e.g., DNS), or one that is translated into another language (e.g., MNSIq) may be preferable, particularly in light of the largely similar resulting AUCs and high correlations amongst the instruments. Future studies will be needed to determine if these questionnaires have differential diagnostic characteristics in different populations, such as populations without obesity.

Although neurologic history and examinations remain the best way to diagnose PN in the clinical setting, PN questionnaires may be useful as clinical screening instruments. The potential of these questionnaires is highlighted by the fact that they have comparable diagnostic characteristics to confirmatory tests such as NCS and IENFD [11, 12]. Currently, PN is often undiagnosed in routine clinical practice; therefore, a patient‐facing questionnaire may alert physicians of the need for further evaluation [28, 29]. In addition, a clinical PN diagnosis requires a neurologic exam, which may be difficult as primary care providers are often not comfortable with neurologic examinations, and wait times and travel burden for patients to see neurologists continue to increase. Specifically, patients waited a median of 34 days in 2019 to see a neurologist after referral (an increase from a median of 21 days in 2012), with many patients waiting several months to see a neurologist [30]. Moreso, there is a disproportionate travel burden among those that are referred to neurologists, as patients in regions with fewer neurologists often drive further distances to receive care [31]. PN questionnaires could identify patients most in need of a neurologist and reassure others that do not. Interestingly, we found the optimal diagnostic threshold according to Youden's *J* index for MNSIq was lower than the previously established threshold [9], while the threshold for DNS was higher [8]. The previously established diagnostic thresholds were determined in cohorts of persons with diabetes, whereas the present study came from a population with obesity. Thus, while there were slight differences in the diagnostic cutoffs in the present study and those in cohorts with diabetes, these differences may not be systematic. Future studies are needed to determine if these threshold differences persist in a larger population and whether the use of PN questionnaires reduces underdiagnosis of PN and subsequently improves neuropathy outcomes.

Strengths of the present study include the comprehensive assessment of PN.

However, our study power is limited by its sample size. In addition, this study only included participants with obesity who were recruited from surgical weight loss clinics, preventing generalizability to broader populations. Furthermore, the median time between completion of the 24‐month outcome measurements at the end of the initial randomized controlled trial and questionnaire completion was 2.1 years, introducing the risk of disease progression bias. Although a shorter follow‐up time would have been preferred, neuropathy outcomes are unlikely to change dramatically during this timeframe. In addition, incorporation bias is possible between the questionnaires and mTCNS given that they both measure neuropathy symptoms [32]. However, given the comparable diagnostic accuracy for mTCNS‐defined PN and other outcomes, it is unlikely that this correlation is substantially affecting the diagnostic accuracy of these questionnaires and PN. Finally, the questionnaires were administered in the same order for all participants, which could have resulted in a training effect and improved accuracy as the questionnaires were completed.

To conclude, we found that patient‐completed questionnaires have good diagnostic accuracy when classifying individuals according to several PN outcomes. E‐PSS had either higher or similar predictive performance compared to the other questionnaires across multiple PN definitions, and further research with larger sample sizes may help elucidate whether these differences are significant. All three questionnaires can be used in epidemiologic studies with the choice of instrument based on factors such as length, ease of interpretation, and whether it has been translated into the required language. Future studies are needed to determine the role of these instruments as clinical screening instruments.

## Author Contributions

N.H. was involved in the study design, performed the statistical analysis and interpretation of the statistical analysis, and wrote the manuscript. E.L.R. was involved in the study design and interpretation of the statistical analysis and provided critical revisions of the manuscript. F.K., L.M., M.H., N.E.T., and P.A.D. provided critical revisions of the manuscript. B.C.C. was involved in the study design, and interpretation of the statistical analysis and provided critical revisions of the manuscript.

## Funding

This study is supported by the NIH NIDDK (R01DK115687). Evan L. Reynolds is supported by the NIH NIDDK (R00DK129785).

## Ethics Statement

This study was approved by the University of Michigan Medical School Institutional Review Board. All study participants provided written informed consent.

## Conflicts of Interest

Nicholas Heaton reports no disclosures. Brian C. Callaghan consults for Dynamed, performs medical legal consultation including the Vaccine Injury Compensation Program, and receives research and editorial support from the American Academy of Neurology. The other authors declare no conflicts of interest.

## Data Availability

The data that support the findings of this study are available from the corresponding author upon reasonable request.
